# Comparative Efficacy of Meloxicam and Placebo in Vasospasm of Patients with Subarachnoid Hemorrhage 

**Published:** 2015

**Authors:** Seyed Mohammad Ghodsi, Niayesh Mohebbi, Soheil Naderi, Mousareza Anbarloie, Ahmad Aoude, Seyed Sohail Habibi Pasdar

**Affiliations:** a *Department of Neurosurgery, Tehran University of Medical Sciences, Tehran, Iran. *; b *Department of Clinical Pharmacy, Faculty of Pharmacy and Research Center for Rational Use of Drugs, Tehran University of Medical Sciences, Tehran, Iran. *; c *Department of Internal Medicine, University of Arizona, Arizona, United States of America.*

**Keywords:** Cerebral vasospasm, Subarachnoid haemorrhage, Anti-inflammatory drugs, Meloxicam

## Abstract

Cerebral vasospasm considered to be a serious cause of morbidity and mortality following subarachnoid haemorrhage (SAH).Despite several available therapeutic options, current protocols do not prevent major consequences of vasospasm. Inflammation is believed to play an important role in post-haemorrhagic vasospasm. Meloxicam is a non-steroidal anti-inflammatory drug. The aim of this study was to compare the efficacy of meloxicam versus placebo on vasospasm in patients with SAH. In this randomized, double-blind, placebo-controlled trial, SAH patients randomly received 7.5 mg meloxicam or placebo twice daily for 7 days. End points were, middle cerebral artery velocity obtained with transcranial doppler, in-hospital mortality, hospital stay and discharge Glasgow Outcome Scale. Eighty-one patients enrolled in the study. (40 received meloxicam, 41 received placebo). Baseline characteristics were similar between the groups. There were no differences in length of hospitalization (17.4 ± 3.1 vs 18.6 ± 4.2 days; p = 0.145), in-hospital mortality rate (15 vs 22%; p-value=0.569), or GOS (p = 0.972) between the two groups. MCA velocity were slightly less in patients who had received meloxicam, but not to a significant degree (p-value=0. 564(. No side effect has been detected for meloxicam. This study did not prove meloxicam efficacy in vasospasm of SAH patients. But it demonstrated that clinical trial of meloxicam in these patients is feasible and probably safe. The effectiveness of meloxicam on cerebral vasospasm has to be studied in larger trials.

## Introduction

Subarachnoid haemorrhage (SAH) although responsible for only 3% of strokes, reported to have titanic fatality (32-67%) (1). Furthermore, about 20% of the survived patients would became disabled, and remain dependent (2). Cerebral vasospasm in SAH patients is one of the main reasons for poor outcome leading to death and disability (3-5). Therefore prevention and treatment of cerebral vasospasm is critical in the management of SAH patients. 

As a matter of fact, precise mechanism of cerebral vasospasm is not fully understood, and current therapeutic protocols do not show sufficient therapeutic effects (6-9). Thus, effective therapeutic strategies also remain to be established. Experimental studies suggest a multifactorial pathogenesis involving leukocyte-mediated inflammation, endothelial injury, NO depletion, and microvascular dysautoregulation (10-13). Many studies have pointed to the role of inflammation as a critical factor in the development of cerebral vasospasm (12-16).

Cyclooxygenase (COX) is an enzyme that catalyses the conversion of arachidonic acid to prostoglandins (PGs), which are considered to be important inflammatory mediators (17).

The effects of anti-inflammatory agents such as methlyprednisolone, cyclosporine, ibuprofen, indomethacin, meclofenamate and piroxicam on cerebral vasospasm have previously been studied (18-25). The outcomes of these studies have been equivocal; in particular, the efficacies observed for Non-steroidal anti-inflammatory drugs (NSAIDs) are mixed and dependent on the study and agent. 

Meloxicam is a NSAID derived from enolic acid that selectively inhibits cycloogenase-2 (COX-2) over COX-1 during prostaglandin synthesis from arachidonic acid (26,27). In an experimental study examining the effect of meloxicam on experimentally induced vasospasm of the rat femoral artery, the dose of meloxicam was 2 mg/Kg intraperitoneally daily. No side effects, including gastrointestinal disturbances, were detected in the rats. Results demonstrate a relationship between the administration of meloxicam and the prevention of vasospasm in a rat model. Meloxicam prevented the development of chronic vasospasm following experimental SAH (28).

To the best of our knowledge there has been no published clinical trial of meloxicam in SAH. This study designed to compare the efficacy of meloxicam versus placebo on vasospasm in patients with SAH.

## Experimental

*Methods*

We conducted a randomized, double blind, placebo controlled clinical study on patients with diagnosis of SAH documented by Computed Tomography (CT) scan who admitted to neurosurgery and neurology wards of Shariati hospital (Tehran, Iran). 

Exclusion criteria was as fallowing: Any contraindications for meloxicam use (sensitivities to meloxicam, aspirin, or any other NSAIDs sensitivity); Serious adverse reaction induced by meloxicam; Long term use of meloxicam or any other NSAIDs before the study; Rebleeding in patient leading to emergency neurosurgical operation; Serious interaction between patient's medications and meloxicam; Unstable hemodynamic condition in patient making doppler sonography impossible. 

All SAH patients admitted to Shariati hospital between April 2011 and February 2013, were enrolled in the study. Patient or the closest family member in charge of the patient (if patient was unconscious) signed the written informed consent. Study protocol, methods of data collection and analysis were approved by the research committee of the Tehran University of Medical Sciences (TUMS). Our clinical trial had been registered in Australian and New Zealand Clinical Trials Registry (ANZCTR) with a registration ID of “ACTRN12613000767707”.

At the time of admission, SAH patients were randomly assigned to one of drug or placebo group. Patients received 7.5 mg meloxicam oral tablets or placebo twice daily for 7 days. All other standard treatments have been performed for all the patients, including initiation of nimodipine 60 mg every four hours for 21 days, appropriate fluid therapy, phenytoin administration, and surgery and clipping for aneurysms. Patients’ demographic data (age, sex, history of Diabetes mellitus (DM), Hypertension (HTN), Coronary artery disease (CAD), SAH, and smoking), physical exam, and Hunt and Hess(H & H) score were recorded.

The study end points were: middle cerebral artery (MCA) velocity obtained with transcranial Doppler (TCD), in-hospital mortality rate, length of hospital stay (number of nights patients spent at the hospital), Glasgow Outcome Scale (GOS) score at discharge day. MCA velocity (V_MCA_ ) measurements by TCD were used as the indicator of vasospasm. V_MCA_ above 120 cm/sec defined as existence of vasospasm, and more than 200 cm/sec defined severe vasospasm. To exclude hyperemic condition, cervical internal carotid artery velocity (V_ICA_ ) were measured, and Lindegaard Ratio calculated [ Lindegaard Ratio = V_MCA_/V_ICA_ ]. Lindegaard Ratio should be above 3 to fulfill vasospasm definition criteria. TCD performed before meloxicam or placebo started for baseline, 4 days and 7days after (fallowing last dose) drug administration. The clinical outcome evaluated by GOS at discharge day for each patient.

All the statistical analysis were performed by means of SSPS version 17 (SPSS Inc., Chicago, IL, USA) and significance was defined as p-value under 0.05. Parametric data were reported as means ± standard deviations (SDs) and student t-test was used, for comparing quantitative variables between the groups. The Mann-Whitney U-test, a nonparametric statistic test, used to assess whether two independent groups are significantly different from each other, and nonparametric data were expressed as medians. Multivariate regression used to evaluate the relationship between independent variables and dependent variables.

## Results

During the study period 81 patients [49 males (60.5%), 32 females (39.5%)] met the inclusion-exclusion criteria for final analysis. Eligible patients were aged 50.2 ± 8.8 years (mean ± SD), range from 26 to 71 years. After randomization 40 patients received meloxicam for 7 days, and 41 patients received placebo, beside standard cares. Thirty-three patients reported to have HTN, and 12 patients with DM, 6 patients had coronary artery disease, and one patient had SAH history. Thirty-one patients were smokers. Eighty-nine aneurysms have seen in patients. Mean ± SD for aneurysm size was 8.1± 1.4 mm [Drug group: (8.5±1.1), Placebo group: (7.6±1.9)]. Thirty-nine carotid bifurcation aneurysms (43.8%), 26 anterior communicating artery aneurysms (29.2%), 19 middle cerebral artery aneurysms (21.35%), 3 anterior cerebral artery aneurysms (3.4%), 2 posterior communicating artery aneurysms (2.2%) perceived. There were no statistically significant differences among the groups in demographic data, and baseline characteristics. The median admission H & H grade was 2. Distribution of H & H grade in both groups exhibited in Table 1. There was no statistically significant difference among the groups for H & H grading. 

**Table 1:** Hunt and Hess grade stratified by meloxicam treatment

**p-value**	**Placebo group (%)**	**Meloxicam group (%)**	**Hunt and Hess grade **
0.972	6(14.6)	8(200)	1
	13(31.7)	12(300)	2
	11(26.8)	11(27.5)	3
	6(14.6)	5(12.5)	4
	5(12.2)	4(10.0)	5

Length of hospital stay was 18.00 ± 3.7days in general, in Melocxicam group 17.4 ± 3.1 days, and for placebo group, it was 18.6 ± 4.2 days. Statistical analysis showed no significant difference demonstrated among the groups (p-value =0.145).

Mortality during hospitalization occurred in 15 patients (18.5%), 6 patients (15.0%) in melocxicam group and 10 patients (21.95%) in placebo group. Again no statistically significant differences among the groups existed (p-value=0.569).

Vasospasm occurred in 13 (32.5%) of melocxicam group, and 18 (44.0%) patients of placebo group based on MCA blood velocity in TCD (*i.e*. at least one of the three measurements higher than 120 cm/sec). Lindegard ratio were higher than 3 in all cases. Although the rate was lower in meloxicam group, there was no statistically significant difference among the drug and placebo groups (p-value=0. 564). 

The median GOS at discharge day from hospital was 3. GOS of patients in both groups demonstrated in Table 2. The difference in GOS between meloxicam and placebo group were not significant (p-value=0.618).

 No patients experienced significant elevations in liver transaminases or creatine phosphokinase levels at baseline or after initiation of statin therapy that required termination of therapy.

**Table 2:** Glasgow outcome scale stratified by meloxicam treatment

** p-value**	**Placebo group (%)**	**Meloxicam group (%)**	**Glasgow Outcome Scale **
0.618	9(22.0)	6(15.0)	1
	14(34.1)	11(27.5)	2
	9(22.0)	15(37.5)	3
	6(14.6)	6(15.0)	4
	3(7.3)	2(5.0)	5

In multivariate regression analyses, for MCA velocity, age and sex showed significant statistical difference; as age progressed MCA velocity reduction was less, and in females MCA velocity reduction was less than males. Regarding Hunt and Hess grade, hypertension and smoking was significantly related. H & H score was higher in patient with hypertension, and was significantly lower in smokers.  Regarding GOS, hypertension was significantly related that GOS was lower in patient with hypertension. Regarding Mortality, Sex and smoking was significantly related; so that, mortality was higher in female patients, and was significantly lower in smokers. No statistically significant relation was detected between independent variants and hospital stay. There was just one patient with the history of past SAH, so statistical judgment about this factor was impossible. There was no adverse reaction detected following meloxicam or placebo use in this trail.

## Discussion

After aneurysmal SAH, cerebral vasospasm is still one of the most important contributors to morbidity and mortality. (3-5) Despite the advances in pharmacological and surgical treatment of SAH, there is still no definitive therapy for cerebral vasospasm. Perhaps the reason underlying this paradox is that many pathophysiological pathways are not yet understood (6-9). In recent studies, it has been well demonstrated that inflammation plays a key role in the pathophysiology of cerebral vasospasm and subsequent cerebral ischemia (12-16).

One study published in United States declared that experimental and clinical evidences lead us to target inflammatory and oxidative cascades for reducing the incidence and impact of cerebral vasospasm (29). In an experimental study ibuprofen and high-dose methylprednisolone resulted in reduction in vasospasm in dogs (18). In a study high-dose methylprednisolone demonstrated beneficial effects on vasospasm after subarachnoid hemorrhage (19). In a published study on 2004 local intracranial delivery of controlled-release ibuprofen by means of ethylene-vinyl acetate copolymers prevented cerebral vasospasm in the rabbits (21). Early treatment with high dose methylprednisolone showed positive effects on delayed cerebral ischemia in aneurysmal subarachnoid hemorrhage patients (22).

In a chronic model of cerebral vasospasm in dogs comparing piroxicam, meclofenamate, ibuprofen, aspirin, and prostacyclin efficacy, NSAIDs significantly diminished the incidence of vasospasm. However, piroxicam was the most effective, meclofenamate, ibuprofen, and aspirin also showed improvements. This observation described to be as a result of piroxicam long half-life. Results implied that appropriate NSAID therapy could improve clinical outcome fallowing SAH (25).

A follow-up study on aspirin effects on delayed cerebral ischemia after aneurysmal subarachnoid hemorrhage revealed protective results cerebrovascular ischemic complications in patients who used aspirin before SAH (23). In a randomized, double-blind, placebo-controlled pilot trial on SAH patients 100 mg aspirin suppositories versus placebo, administered instantly after surgical clipping of the aneurysm for 21 days. The result suggested bigger trials to evaluate aspirin efficacy (24). In an experimental study examining the effect of meloxicam on experimentally induced vasospasm of the rat femoral artery, the dose of meloxicam was 2 mg/Kg intraperitoneally daily and no side effects, including gastrointestinal intolerance, were detected in the rats. Results demonstrate a relationship between the administration of meloxicam and the prevention of vasospasm in a rat model. Meloxicam prevented the development of chronic vasospasm following experimental SAH (28).

In our randomized, double blind, controlled trail of 81 SAH patients treated at Shariati hospital, meloxicam group showed better outcome, including: less incidence of cerebral vasospasm, mortality, hospital stay, and also improvement in GOS at discharge in comparison to placebo group. But there was not statistically significant difference among the groups in these parameters. 

Multivariate regression analyses revealed that age and sex had significant effects in MCA velocity changes. In older patients MCA velocity reduction was less, and in females MCA velocity reduction was less than males. More researches required for understanding causality of this detection.

Regarding Hunt and Hess scale, hypertension and smoking had significant relation. Hunt and Hess scale was higher in patient with hypertension. Hypertension proven to be a risk factor for SAH, subsequently this impact was predictable (30). Hunt and Hess grade was significantly lower in smokers. The protective effect of smoking has been demonstrated before (30). There was no significant association related to history of DM and CAD with vasospasm in this study. Regarding GOS, hypertension was significantly related; GOS was lower in patient with hypertension. Hypertension noxious effects were established previously (30). No statistically significant relation was detected between independent variants and hospital stay. In Mortality assessment, sex and smoking was significantly related. Mortality was higher in female patients that controversial evidences exist in this matter. And Mortality was significantly lower in smoker patients. Once more this is another proof for the protective effect of smoking (30). Substances decrease neurotoxicity by inhibiting catecholamine activities or excessive excitatory neurotransmitters can show neuroprotective effects. (31, 32)Meloxicam therapy was well tolerated and did not result in any adverse reaction for patient during the study period. Consequently clinical trials of meloxicam in SAH seem to be viable and probably safe. The effectiveness of meloxicam on functional outcome and delayed cerebral vasospasm has to be studied in larger trials.

## References

[1] van Gijn J, Rinkel GJ (2001). Subarachnoid haemorrhage: diagnosis, causes and management. Brain.

[2] Hop JW, Rinkel GJ, Algra A, van Gijn J (1997). Case-fatality rates and functional outcome after subarachnoid hemorrhage: a systematic review. Stroke.

[3] Mayberg MR (1998). Cerebral vasospasm. Neurosurg.Clin. North. Am.

[4] Kreiter KT, Copeland D, Bernardini GL, Bates JE, Peery S, Claassen J, Du YE, Stern Y, Connolly ES, Mayer SA (2002). Predictors of cognitive dysfunction after subarachnoid hemorrhage. Stroke.

[5] Claassen J, Bernardini GL, Kreiter K, Bates J, Du YE, Copeland D, Connolly ES, Mayer SA (2001). Effect of cisternal and ventricular blood on risk of delayed cerebral ischemia after subarachnoid hemorrhage: the Fisher scale revisited. Stroke.

[6] Belen D, Besalti O, Yiğitkanli K, Kösemehmetoğlu K, Simşek S, Bolay H (2007). Leflunomide prevents vasospasm secondary to subarachnoid haemorrhage. Acta. Neurochir.

[7] Vural M, Cosan TE, Ozbek Z, Cosan D, Sahin F, Burukoglu D (2009). Digoxin may provide protection against vasospasm in subarachnoid haemorrhage. Acta. Neurochir.

[8] Günaldi O, Tuğcu B, Cöllüoğlu B, Güçlü DG, Tanriverdi O, Akdemir H, Bayindir C (2010). Morphometric analysis of the influence of selenium over vasospastic femoral artery in rats. Acta. Neurochir.

[9] Mocco J, Zacharia BE, Komotar R, Connolly ES Jr (2006). A review of current and future medical therapies for cerebral vasospasm following aneurismal subarachnoid hemorrhage. Neurosurg. Focus.

[10] Hansen-Schwartz J, Hoel NL, Zhou M, Xu CB, Svendgaard NA, Edvinsson L (2003). Subarachnoid hemorrhage enhances endothelin receptor expression and function in rat cerebral arteries. Neurosurg.

[11] Zimmermann M, Seifert V (1998). Endothelin and subarachnoid hemorrhage: an overview. Neurosurg.

[12] Crowley RW, Medel R, Kassell NF, Dumont AS (2008). New insights into the causes and therapy of cerebral vasospasm following subarachnoid hemorrhage. Drug Discov. Today.

[13] Dumont AS, Dumont RJ, Chow MM, Lin CL, Calisaneller T, Ley KF, Kassell NF, Lee KS (2003). Cerebral vasospasm after subarachnoid hemorrhage: putative role of inflammation. Neurosurg.

[14] Aihara Y, Kasuya Y, Onda H, Hori T, Takeda J (2001). Quantitative analysis of gene expressions related to inflammation in canine spastic artery after subarachnoid hemorrhage. Stroke.

[15] Fassbender K, Hodapp B, Rossol S, Bertsch T, Schmeck J, Schütt S, Fritzinger M, Horn P, Vajkoczy P, Kreisel S, Brunner J, Schmiedek P, Hennerici M (2001). Inflammatory cytokines in subarachnoid haemorrhage: association with abnormal blood flow velocities in basal cerebral arteries. J. Neurol. Neurosurg. Psychiatry.

[16] Thai QA, Oshiro EM, Tamargo RJ (1999). Inhibition of experimental vasospasm in rats with the periadventitial administration of ibuprofen using controlled-release polymers. Stroke.

[17] Santos AR, Vedana FM, De Freitas GA (1998). Antinociceptive effect of meloxicam, in neurogenic and inflammatory nociceptive models in mice. Inflamm. Res.

[18] Chayatte D (1989). Prevention of chronic vasospasm in dogs with ibuprofen and high-dose methylprednisolone. Stroke.

[19] Yamakawa K, Sasaki T, Tsubaki S, Nakagomi T, Saito I, Takakura K (1991). Effect of high-dose methylprednisolone on vasospasm after subarachnoid hemorrhage. Neurol. Med. Chir.

[20] Chen D, Nishizawa S, Yokota N, Ohta S, Yokoyama T, Namba H (2002). High-dose methylprednisolone prevents vasospasm after subarachnoid hemorrhage through inhibition of protein kinase C activation. Neurol. Res.

[21] Frazier JL, Pradilla G, Wang PP, Tamargo RJ (2004). Inhibition of cerebral vasospasm by intracranial delivery of ibuprofen from a controlledrelease polymer in a rabbit model of subarachnoid hemorrhage. J. Neurosurg.

[22] Chyatte D, Fode NC, Nichols DA, Sundt TM Jr (1987). Preliminary report: Effects of high dose methylprednisolone on delayed cerebral ischemia in patients at high risk for vasospasm after aneurysmal subarachnoid hemorrhage. J. Neurosurg.

[23] Juvela S (1995). Aspirin and delayed cerebral ischemia after aneurysmal subarachnoid hemorrhage. J. Neurosurg.

[24] Hop JW, Rinkel GJE, Algra A, Berkelbach van der Sprenkel JW, van Gijn J (2000). Randomized pilot trial of postoperative aspirin in subarachnoid hemorrhage. Neurol.

[25] White RP, Robertson JT (1983). Comparison of piroxicam, meclofenamate, ibuprofen, aspirin, and prostacyclin efficacy in a chronic model of cerebral vasospasm. Neurosurg.

[26] Engelhardt G, Bogel R, Schnitzler C, Utzmann R (1996). Meloxicam: influence on arachidonic acid metabolism Part II. In-vivo findings. Biochem.. Pharmacol.

[27] Ogino K, Hatanaka K, Kawamura M, Katori M, Harada Y (1997). Evaluation of pharmacological profile of meloxicam as an anti-inflammatory agent, with particular reference to its relative selectivity for cyclooxygenase-2 over cyclooxygenase-1. Pharmacol.

[28] Hakan T, Berkman MZ, Ersoy T, Karataş I, San T, Arbak S (2008). Anti-inflammatory effect of meloxicam on experimental vasospasm in the rat femoral artery. J. Clin. Neurosci.

[29] Lehmann E, Sagher O (2008). Novel treatments for cerebral vasospasm following aneurysmal subarachnoid hemorrhage. Acta. Neurochir. Suppl.

[30] Spears J, Macdonald RL, Weir B, Winn HR (2011). Preioperative Management of Subarachnoid Hemorrhage.

[31] Moshtaghie M, Malekpouri P, Saeed-zadeh M, Messripour M, Moshtaghie AA (2013). Catecholamine contents of different region of adult rat brain are altered following short and long-term exposures to Pb+2. Iran. J. Pharm. Res.

[32] Salavati P, Ramezani M, Monsef-Esfahani HR, Hajiagha R, Parsa M, Tavajohi S, Ostad SN (2013). Neuroprotective effect of total and sequential extract of scrophularia striata boiss in rat cerebellar granule neurons following glutamate- induced neurotoxicity: an in-vitro study. Iran. J. Pharm. Res.

